# Anesthetic Management of a Patient With Subglottic Stenosis: The Crucial Role of Communication Between Teams

**DOI:** 10.7759/cureus.62250

**Published:** 2024-06-12

**Authors:** Dulce Pereira, Ana S Cruz, Luís Dias, Cristina Gomes

**Affiliations:** 1 Anesthesiology, Centro Hospitalar de Tondela-Viseu, Viseu, PRT; 2 Anesthesiology, Hospital de Braga, Braga, PRT; 3 Otorhinolaryngology and Head and Neck Surgery, Hospital de Braga, Braga, PRT

**Keywords:** bardet-biedl syndrome (bbs), multidisciplinary approach, jet ventilation, fibroscopy, airway management, subglottic stenosis

## Abstract

Subglottic stenosis is characterized by the narrowing of the airway at the inferior edge of the cricoid cartilage level. It is either congenital or acquired, the latter being more commonly secondary to internal iatrogenic trauma. Airway management of these cases is challenging and requires multidisciplinary discussion. We present a case of a 17-year-old boy scheduled for tracheostomy in the context of subglottic stenosis probably caused by prolonged endotracheal intubation. On the day of surgery, it was decided to perform an asleep fiberoptic visualization of the lesion through a supraglottic device, which revealed a narrow circumferential fibrous membrane just below the vocal cords. Given the findings, a suspension laryngoscopy accompanied by supraglottic manual jet ventilation was performed. Balloon dilatation with the application of mitomycin C was the elected otorhinolaryngologic technique. At the end of the procedure, a fiberoptic exam was performed and only a minimal portion of the membrane remained. The patient was asymptomatic on follow-up visits. We aim to raise awareness of how the anesthetic management of patients with subglottic stenosis may prove challenging. Communication between anesthetic and surgical teams is essential for the achievement of the main goal, which is the acquisition of an adequate airway that allows normal patient activity associated with minimal postoperative morbidity.

## Introduction

Subglottic stenosis is defined as a stricture in the area extending from the lower surface of the true vocal cords to the inferior surface of the cricoid cartilage [1]. The etiology includes a diverse group of congenital or acquired conditions which can be malignant or non-malignant and cause significant morbidity and mortality. Acquired subglottic stenoses are the most common type. It can be caused by external injury to the anterior part of the neck or internal laryngeal trauma caused by tracheal intubation or tracheostomy. The pathophysiology of this type of acquired subglottic stenosis involves exaggerated cuff pressure, which causes edema, ischemia, and ulceration of the area [2,3], and then the cartilage shows necrosis and collapses. Healing of the affected cartilage is by secondary intention and deposition of fibrous tissue [4]. The result is a firm scar similar to healing events in the skin, equivalent to a “hypertrophy scar” [1]. Incidence has decreased significantly in recent decades due to improved cuff design and regular cuff pressure monitoring after intubation [5]. Other less frequent acquired causes include laryngeal burns, post-radiotherapy status, chronic infection, chronic inflammatory diseases, tumors, and idiopathic. Recent research suggests the role of a dysregulated T cell-specific immune response leading to collagen and extracellular matrix deposition by fibroblast and consequently to idiopathic subglottic stenosis [6].

Its management varies greatly depending on the underlying condition. There are, however, common targets such as treatment of the underlying etiology, restoration of airway patency, and symptom relief. A multidisciplinary approach and communication involving anesthesiologists and otorhinolaryngologists is extremely important for the evaluation and discussion of treatment options for this highly complex airway disease, improving patients’ outcomes and diminishing the respective morbidity.

In this case report, we describe the alternative management of a known acquired symptomatic subglottic stenosis previously scheduled for tracheostomy and outline the crucial role of communication between teams.

## Case presentation

A 17-year-old boy (height: 170 cm; weight: 94 kg; body mass index: 32,5 kg/m^2^), with American Society of Anesthesiologists (ASA) physical status classification III, was scheduled for tracheostomy in the context of subglottic stenosis.

**Figure 1:**
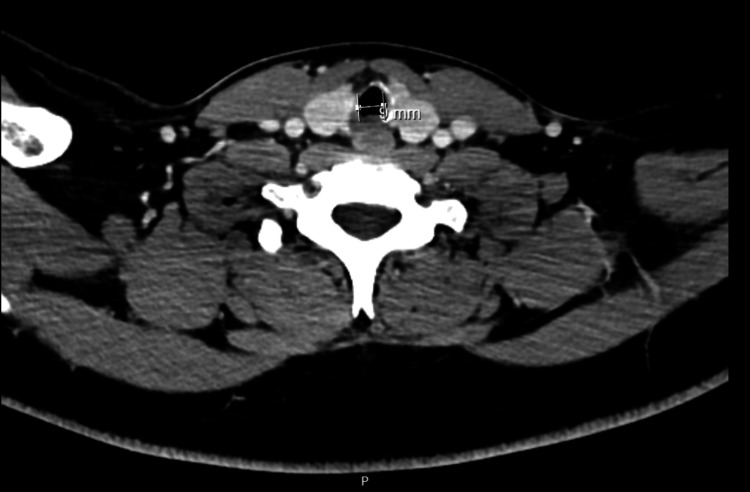
Axial cervicothoracic CT scan image showing a subglottic formation and a minimum diameter of 9 mm in the subglottic larynx

Approximately six months before surgery, he was admitted to a pediatric intensive care unit because of traumatic brain injury with subarachnoid hemorrhage and cerebral contusion. He was under mechanical ventilation/endotracheal intubation for five days, which was the most probable cause for the referred subglottic stenosis. Other relevant previous medical history included Bardet-Biedl syndrome and asthma without recent exacerbations motivating hospital admission. His regular medication included budesonide, formoterol, fenofibrate, and montelukast. He had no medication allergies. Preoperative airway physical examination revealed a grade II Mallampati score, mouth opening greater than 3 cm, thyromental distance of 6 cm, good cervical spine mobility, and a slightly widened cervical perimeter. He mentioned shortness of breath with exertion but during pre-anesthetic evaluation, he was asymptomatic, at rest. Preoperative laboratory results (hemogram, renal function, and coagulation) were unremarkable. Cervicothoracic computed tomography (CT) scan showed a subglottic polypoid formation, 13.5 mm away from the inferior margin of the thyroid cartilage, measuring approximately 3.5 mm in maximum extension and another contralateral polypoid area, very likely consisting of fibrous tissue, with a minimum diameter of 9 mm in the subglottic larynx (Figure 1).

**Figure 2:**
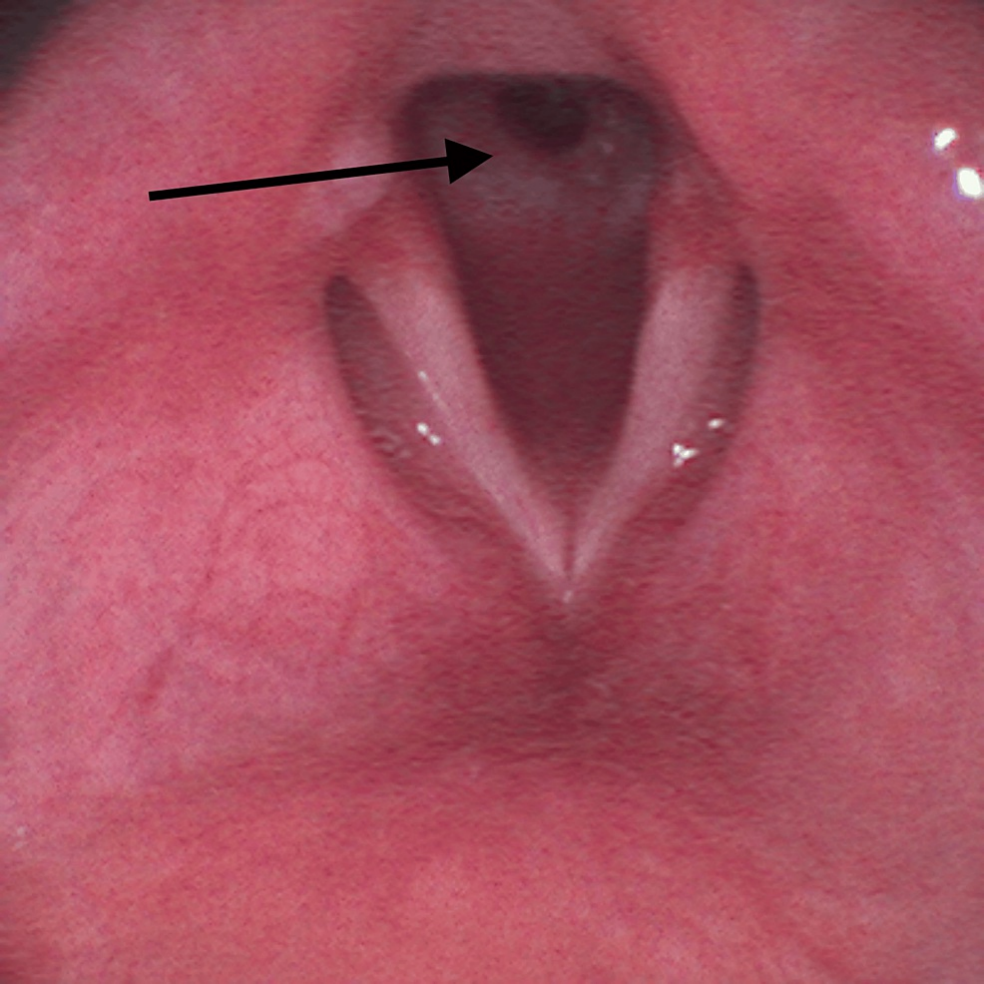
Circumferential subglottic fibrous membrane (arrow) observed during asleep fiberoptic visualization

On the surgery day, a reassessment of his airway management plan was performed by a multidisciplinary team including anesthesiologists and otorhinolaryngologists. It was decided to make an asleep fiberoptic visualization of the lesion through a supraglottic device. After adequate pre-oxygenation, endovenous induction was performed using a target-controlled infusion (TCI) of propofol, Schneider model with target effect-site concentration of 4 µg/mL, and of remifentanil, Minto model. A dose of 60 mg of rocuronium was administered and a supraglottic device, AuraGain® size number four was introduced without trauma. Total intravenous anesthesia was maintained using the referred TCI. While maintaining volume-controlled ventilation with a maximum inspiratory pressure of 20 cmH_2_O, an Ambu® aScope™ 4 Broncho Slim 3.8/1.2 (Ambu A/S, Ballerup, Denmark) was introduced through the laryngeal mask and a circumferential fibrous membrane just below vocal cords was observed (Figure 2). 

Controlled ventilation was maintained uneventfully during all the procedures and peripheral oxygen saturation (SpO_2_) levels were maintained at 99-100%.

**Figure 3:**
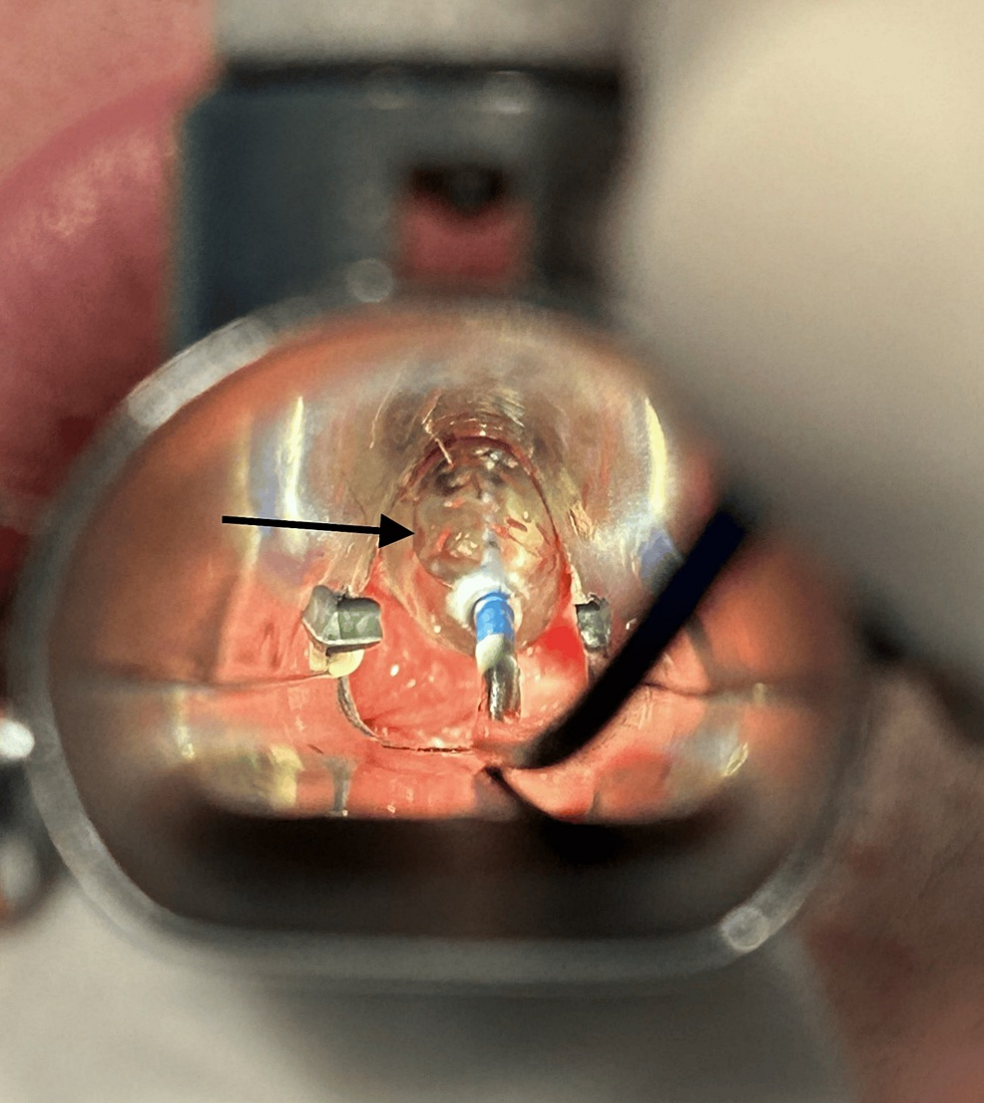
Balloon dilatation (arrow) of the subglottic stenosis

**Figure 4:**
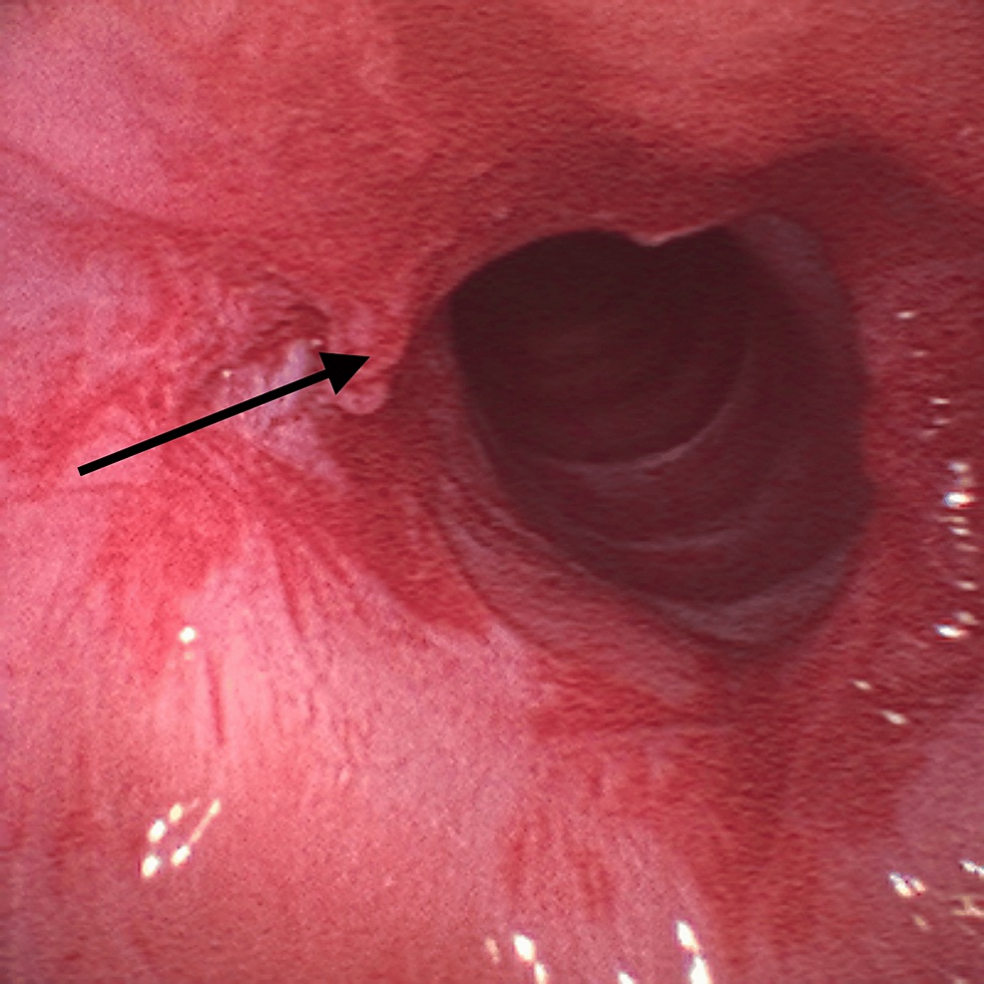
Minimal portion of the membrane remaining at the end of surgery (arrow)

Given the findings, the supraglottic device was removed and the patient was placed in suspension laryngoscopy. Supraglottic manual jet ventilation was applied using a narrow-bore, noncompliant cannula attached to the rigid bronchoscope at one of the accessory ports on the head of the instrument. A maximum tracheal pressure of 35 cmH_2_O and a jet frequency of 10 per minute allowing adequate time for exhalation via passive recoil of the lung and chest wall preventing air trapping and barotrauma were applied. It was also performed with careful observation of the patient’s chest movements to ensure adequate tidal volumes without overdistension. Balloon dilatation with the application of mitomycin C was the elected otorhinolaryngologic technique (Figure 3) and at the end of the procedure, only a minimal portion of the membrane was noticed (Figure 4).

No significant events were observed. Hemodynamic stability was maintained during the two-hour surgery. At the end of the procedure, a supraglottic device was introduced, and a new fiberoptic visualization through the laryngeal mask was performed. The effectiveness of the treatment was verified - only a minimal portion of the membrane remained. The residual muscle relaxation was reversed using sugammadex and anesthetic emergence was uneventful. The patient was subsequently admitted to the post-anesthetic care unit for three hours and then transferred to the ward, uneventfully.

The patient was discharged home after two days of surgery without any complications. No symptoms were observed in follow-up visits, and he was able to perform physical activity on the 37th day of the post-surgical procedure.

## Discussion

Anesthetic management of patients with subglottic stenosis is challenging since there is a central airway obstruction associated with the permanent sharing of the airway with the surgeon during the intraoperative period.

The surgical management of these lesions ranges from observation with supportive care in times of exacerbations (fiberoptic nasopharyngoscopy, bronchodilators, and antireflux medications) to endoscopic repair, dilatation, CO_2_ laser, or complicated open surgical reconstructions of the patient’s airway [7]. The major goal is the acquisition of an adequate airway to allow the patient’s normal activities without any requirement for tracheostomy.

Anesthesiologists must weigh up the benefits and risks of the chosen technique for the airway approach [8]. A variety of airway management techniques has been described [9,10]. However, determining which technique is associated with fewer complications or better outcomes is difficult since subglottic stenosis is relatively rare and studies scarce [11] with slight performance differences related [12]. Anesthetic techniques supporting endoscopic surgery for subglottic stenosis repair should provide the surgeon with a clear, immobile field and the anesthesiologist with the possibility of minimal ventilation, oxygenation, and hemodynamic compromise [13].

## Conclusions

Subglottic stenosis can present a challenge regarding airway management. It is essential to perform a careful anamnesis, physical examination, and characterization of the respective extent and severity. The ideal surgical approach depends on the severity of the disease, the shared surgeon and anesthesiologist’s choice, and perioperative capabilities. In the past, minimally invasive treatment has proven to be efficient. However, for high-grade stenosis, major surgeries that include laryngotracheal resection or cricotracheal resection remain options. Anesthesia in this context is challenging. Therefore, large multicentric studies should be developed to determine the type of airway management associated with better outcomes.
